# Creating a Practical Transformational Change Management Model for Novel Artificial Intelligence–Enabled Technology Implementation in the Operating Room

**DOI:** 10.1016/j.mayocpiqo.2022.09.004

**Published:** 2022-10-27

**Authors:** Tianqi G. Smith, Hamid Norasi, Kelly M. Herbst, Michael L. Kendrick, Timothy B. Curry, Teodor P. Grantcharov, Vanessa N. Palter, M. Susan Hallbeck, Sean P. Cleary

**Affiliations:** a Robert D. and Patricia E. Kern Center for the Science of Health Care Delivery, Mayo Clinic, Rochester, MN; b Health Care Delivery Research, Mayo Clinic, Rochester, MN; c Department of Surgery, Mayo Clinic, Rochester, MN; d Department of Anesthesiology, Mayo Clinic, Rochester, MN; e Department of Surgery, University of Toronto, Toronto, Canada; g International Centre for Surgical Safety, Toronto, Canada; h Li Ka Shing Knowledge Institute, St. Michael’s Hospital, Toronto, Canada

**Keywords:** AI, artificial intelligence, CM, change management, FAQ, frequently asked questions, IRB, Institutional Review Board, IT, information technology, OR, operating room, PM, project manager

## Abstract

**Objective:**

To identify change management (CM) strategies for implementing novel artificial intelligence and similar novel technologies in operating rooms and create a new CM model for future trials and applications inspired by the abovementioned strategies and established models.

**Methods:**

Key phases of technology implementation were defined, and strategies for transformational CM were created and applied in a recent CM experience at our institution between October 15, 2020 and October 15, 2021. We appraised existing CM models and propose the newly created model.

**Results:**

The key phases of the technology implementation were as follows: (1) team assembly; (2) committee approvals; (3) CM; and (4) system installation and go-live. Key strategies were (1) assemble team with necessary expertise; (2) anticipate potential institutional cultural and regulatory hurdles; (3) add agility to project planning and execution; (4) accommodate institutional culture and regulations; (5) early clinical partner buy-in and stakeholder engagement; and (6) consistent communication, all of which contributed to the new CM model creation.

**Conclusion:**

Key CM strategies and a new CM model addressing the unique needs and characteristics of operating room novel technology implementation were identified and created. The new model may be customized and tested for individual institution and project’s needs and characteristics.

The operating room (OR), like the health care industry in general, is subject to constant and unpredictable changes.^1^ Reasons for these changes include efforts to drive improvement in care quality and clinical outcomes, patient safety, teamwork efficiency, technological advancement, and policy changes, among others. The changes can be classified as (1) developmental changes that are incremental and nonfundamental; (2) transitional changes that achieve a known desired state from an existing one; and (3) transformational changes that are radical and result in the creation of an organization that continuously learns, adapts, and improves.^2^ Managing change, especially transformational change, takes significant and concerted effort from leadership and staff to navigate unpredictability and resistance.^3^

In this article, we present a case of recent transformational change management (CM) in the ORs of our institution that did not fit the existing CM models (Table 1),^3^, ^4^, ^5^, ^6^, ^7^, ^8^, ^9^, ^10^, ^11^, ^12^, ^13^, ^14^, ^15^, ^16^, ^17^, ^18^, ^19^^,^^20^^,^^21^ which were identified from a narrative literature search.^2^, ^3^, ^4^, ^5^, ^6^, ^7^, ^8^, ^9^, ^10^, ^11^, ^12^, ^13^, ^14^, ^15^, ^16^, ^17^ The lack of fit was partly because many existing tools were not geared toward organizational or transformational change but instead focused on individual, developmental, or transitional changes. Additionally, no model was specific enough as a practical journey map yet generic enough to apply to this study’s novel technology CM process in the OR.

**Table 1:** Some Existing CM Models in Relation to Our Project and the HNT CM Model

CM Model	Definition	What Fell Short of Our Needs?	How does the HNT CM Model Contribute?
ADKAR	Five “practical” steps for individual or organization level change: Awareness, Desire, Knowledge, Ability, and Reinforcement (ADKAR).^3^	For our goal, a more complex CM model that considers organizational culture and is less prescriptive and more agile was required.	The HNT CM model considers a key task force on CM, the project team of core and ad hoc members, and the institutional culture and regulations for customization.
Bridges Transition Model (BTM)	The 3-stage (endings, neutral zone, new beginnings) Bridges Transition Model is based on individuals’ inner psychological process (known as transition) and the support that people need through the change.^4^ It helps manage the human side of change.	The BTM’s scope is limited as it emphasizes human transition. It is not a comprehensive organizational strategy for CM and was therefore not appropriate to be used independently for our study.	The HNT CM model provides a process framework that provides strategic guidance for both human and organizational transitions.
General Electric (GE) Change Acceleration Process Model (CAP)	The GE’s CAP model is used for speeding up the transition state and facilitating a successful organizational change. It emphasizes central leadership and considers the importance of cultural factors. It's 7 steps: Leading Change, Creating a Shared Need, Shaping a Vision, Mobilizing Commitment, Making the Change Last, Monitoring Progress, Changing Systems and Structures, enable a sustainable change.^5^	Our project required joint leadership from practice, research, and administration. The CAP model’s central leadership focus could not capture the complexity of our change.	Besides concerted leadership efforts, navigating and adhering to institutional culture and regulations, eg, acquiring committee approvals and considering unionized staff’s perspectives in our case, were crucial CM strategies that the HTN CM model provides to guide CMs that are complex, transformational, and novel-technology driven.
Jick’s Model	Jick’s model is a 10-principle pragmatic tactical approach that highlights the fact that implementing change is a dynamic and continuing process in which the strategy and vision of change are its starting points. The change itself and how it is implemented are both important. Jick advised the organizations that are implementing change to overcome the many challenges they would face by developing practical organizational solutions.^6^^,^^7^	Despite our CM aligning with Jick’s 10-principle approach and the model identifying change as a continuous process, it neither offered expectation or guidance on resolving unpredictable challenges in the process nor provided counsel regarding interdepartmental collaboration to accomplish complex changes.	The HNT CM model identified understanding and predicting challenges as a formal step of CM, its sequential relationship with team assembly and agile planning and execution, and its parallel relationship with accommodating institutional culture and regulations, clinical partner buy-in, and consistent communication. The layout of the model intends to raise the readers’ awareness of the complexity of organizational and transformational changes and to inspire case-driven thinking of applying each provided strategy.
Kotter’s Theory	Kotter’s theory introduces an 8-step process for leading organizational transitional change.^2^ Its core belief of leadership’s importance in creating and sustaining changes, the importance of employees’ engagement, and its stepwise approach were also used in this project’s implementation.^8^, ^9^	Steps such as “develop and form a strategic vision” neither informs possible unpredictability in the process nor guides the user to consider individual institution policy and culture.	Same as above due to the overlap of Kotter’s Theory and Jick’s Model.^33^
Lewin’s Model	The 3-step Lewin’s model “Unfreezing, Change, Refreezing” provides sequential anchors for a somewhat linear systemic change.^10^	Despite a universally applicable model, the broad steps of this model vary depending on institutional situations and interpretations.^10^^,^^11^ It does not offer a direct and structural journey map for CM that our project underwent.	Broadly complying with the 3-step Lewin’s model, the HNT CM model provides a more zoomed-in strategic CM journey map within the health care context.
McKinsey 7-S Model	The McKinsey 7-S model describes the importance of the interaction between 7 organizational elements (Strategy, Structure, Systems, Skills, Staff, Style, Shared values)^12^ in organizational changes for enhanced effectiveness.	When the interconnected 7-S’s in the McKinsey model are aligned, orchestrated changes are under way. However, the model itself does not entail ways to align the s’s.^12^ This model has been mentioned to be complex and not easy to use in a large organization.^13^ It is often used to analyze but not to manage changes within an organization.	The HNT CM model intends to map a followable path with strategies that can be applied with customization.
Nudge Theory	The Nudge Theory is an application of behavioral economics that uses a choice structure that positively reinforces people’s behavior without forbidding their freedom of choice or imposing mandatory obligations. It has found application among health care professionals in the clinical setting, although requires additional research. ^14^	The Nudge Theory can be useful when assisting to achieve the desired outcome unnoticeably when applied in combination with a more enforceable model (eg, the HNT CM model), to facilitate adherence.	The HNT CM model is intended to be used for more structured changes: the changes take place at a planned time and location, and among targeted populations. People have freedom of choice but plan their choices and receive approvals.
PDCA	The iterative PDCA model can be applied to implement changes for improvement. It is applicable to changes in complex projects or smaller and more frequent changes.^15^^,^^16^	Even though it is serving as a helpful implementation guide, it omits the CM process.^15^^,^^16^	The HNT CM model guides CM, often a component of implementation, on an organizational level and a transformational scale.
RE-AIM	The RE-AIM (Reach, Effectiveness, Adoption, Implementation, Maintenance) model has been utilized to plan, evaluate, and review a variety of health promotion and disease management interventions. RE-AIM is only informative if all 5 dimensions are concurrently measured.^17^^,^^35^	RE-AIM was traditionally applied in public health and behavior change research, and has been increasingly applied in clinical, community, and corporate settings. It was developed to assist translation of scientific advances into practice, although has been proven slow and inequitable.^18^^,^^19^ The purpose of RE-AIM is not aligned with our project’s needs: to manage change associated with implementing novel technology in the OR.	Differing from RE-AIM’s requirement of concurrent measurement of all dimensions of the of model, the HNT model intends to guide CMs via a journey map that leads the project teams to focus on one or a few specific aspects of the project at a time and in a sequence. This semi-sequential approach may assist teams to manage the CM with prioritization.

ADKAR, The Awareness, Desire, Knowledge, Ability, and Reinforcement; IT, information technology; OR, operating room; PDCA, plan-do-check-act; RE-AIM, Reach, Effectiveness, Adoption, Implementation, Maintenance.

This project installed an artificial intelligence (AI)–enabled surgical analytical system (OR Blackbox, Surgical Safety Technologies) (the “system”) in 3 ORs of a quaternary research hospital for both practice and research purposes. The system, intended to function not as substitution but as assistance to existing OR technologies, used AI models, computer vision algorithms, and machine learning to capture and analyze patterns of behavior and workflow in ORs, such as surgical instrument uses and staffing levels. The primary objective was to improve practice (care quality and outcomes in surgical patients). Secondary objectives were enhancing OR efficiency, intraoperative teamwork, and postoperative quality conference reviews.

This specific AI–enabled surgical analytical system has been applied to develop diagnostic and predictive intraoperative algorithms.^18^^,^^19^^,^^22^ It improves upon the advantages of current surgical data recording through (1) objective intraoperative data collection, presentation, human performance analysis, and review^18^^,^^23^, ^24^, ^25^; (2) postoperative team debriefing enhancement^18^; (3) improved nontechnical performance analysis^26^^,^^27^; and (4) enhanced understanding of adverse events.^23^ It promises opportunities to automate, synchronize, and ultimately analyze multiple continuous complex data streams (eg, device-related patient data such as vital signs and intraoperative data: surgical site and room audiovisual recordings) arising from the OR environment in short periods of time for purposes such as nonbiased and evidence-based adverse event review and panoramic observation of OR efficiency and teamwork. The automation also reduces human labor intensity^23^^,^^27^^,^^28^ and potential human interruption of existing OR workflow.

Several hospitals have implemented this specific system. It has been shown to be effective in analyzing teamwork and in turn promoting surgical staff’s nontechnical skills, increasing OR efficiency, and ultimately improving patient outcomes.^23^ The system captures audiovisual recordings of the OR and surgical site in addition to patient physiologic parameters, device usage, and environmental data for analysis of surgical safety, quality, efficiency, engagement, and teamwork. To maintain staff and patient privacy and confidentiality, their sex, faces, skin and clothing colors, body shapes, and voices are de-identified from recordings by AI algorithms; personal information mentioned is redacted. De-identified recordings can be requested by the surgical practice for purposes such as adverse event analysis, education, and research for specific cases. Additionally, aggregated reports on OR efficiency and team skills (eg, team engagement) can be generated on all surgical cases.

Attracted to this technology’s potential positive outcomes, the project team at our institution proposed to pilot test the system in 3 ORs. Meanwhile, the team also anticipated that this transformational change would require sustained leadership championing, as well as staff acceptance and adherence, as the technology’s effect on OR workflow, staff workload, and psychology was unknown. Therefore, strategic CM effort was required throughout the implementation process as the project faced unprecedented challenges at our institution. Resistance to change, a human nature, was anticipated.^29^

From the experiences garnered from this project, a practical CM model was created, not only for similar types of future projects at our institution but also for other institutions in the industry that plan to manage similar changes despite different organizational structure and culture,^30^ funding sources, and staff salary structure among hospital environments. The authors identify translatable CM strategies from the key experiences of this CM process and propose a practical CM model derived from this pioneering experience as a journey map to future transformational technology CM in the OR.

## Methods

### Project Implementation Process

The novelty of the systems technology and its implications of patient privacy, data confidentiality, and the institutional internet security requirements, required the project team to strategize the implementation process, especially its CM segment. Our project underwent 4 main implementation phases between October 2020 and October 2021: (1) project team assembly, (2) committee approvals, (3) CM, and (4) system installation and go-live (Table 2). CM strategies and efforts were used in all phases.

**Table 2:** Operating Room (OR) Blackbox Project Implementation Process

Implementation Phases	Descriptions
(1) Project team assembly	The core team was comprised of administrative leaders, surgeons, and research experts. Other team members were brought in when their expertise was required for the ongoing project phases and were dispersed as these phases were complete.
(2) Committee approvals	The project team initially selected to follow the research approval process out of 2 distinct institutional approval routes for new equipment and technology implementation: research and practice. However, the team navigated a hybrid practice and research approval process as the project progressed because of the hybrid nature of the project (eg, data collected by the OR Blackbox would be used for both quality improvement and research purposes). The team worked closely with teams in legal, patient privacy, risk assessment, and information technology to ensure patient and provider privacy, data confidentiality and internet security.
(3) Change management	The team socialized the project among clinical leaders, partners, and OR staff. The team communicated openly and consistently, encouraged “influencers” to advocate for the project, addressed the “resisters” concerns adequately, and prepared for publicity, despite that publicity was not intended.
(4) System installation and go-live	Once de-identified institutional OR device-based patient data from various institutional electronic health record systems were verified to ensure successful system initiation, all equipment was purchased, delivered, and installed in the 3 identified ORs. Information technology experts were engaged for automated data capturing setup, initial system validation and process set up. Sequentially, data collection started and continued 24/7 as soon as the system went live. The first artificial intelligence-generated report was available for viewing by providers, faculty, and staff 2 weeks post-go-live.

### CM Strategies

The CM process of this project was challenged by its implementation environment, the complex and unpredictable adaptive health care system,^31^^,^^32^ the project’s nontraditional practice and research paradigms, and the unique institutional data security and confidentiality protection requirements. Specifically, the project required approval from research committees due to its exploratory motivation for adopting novel technology as well as privacy and data integrity considerations; as data collected by the system are sent to an external server located in a different country for analysis. Practice-related committee requirements such as the quality improvement and surgical safety initiatives that the system promised were also to be satisfied as this project had generated significant interest among clinical practice leaders and stakeholders.

Some committees processed approvals in parallel, whereas others reviewed and approved in sequence. Themes of the committees included budget, OR and IT infrastructure space, human resources, unions, patient rights advocates, surgical and procedural standards and operations, surgical quality, surgical leadership, clinical systems oversight, clinical practice, risk management, equipment and supplies, Institutional Review Board (IRB), legal, contracting, data security and privacy, and internet security.

Additionally, OR staff resistance to data recording systems^18^^,^^23^, ^24^, ^25^ was anticipated as the detailed and comprehensive level of data capture could appear intimidating to staff despite the widespread strict clinical oversight of the ORs and existing camera systems. Managing, socializing, and communicating the change consistently among various groups of the OR staff (eg, surgical, anesthesia, and nursing), of which select groups are unionized, were also a challenge.

To address these challenges, the project team developed several key strategies that contributed significantly to the project’s CM outcome. We summarized the key points for each strategy and compiled these key points into a checklist (Table 3).^33^ Each item in the checklist can be used as a reminder for future CM project teams when considering specific aspects of the CM at specific time points and/or be used as a base to customize on when navigating their own complex CM process. This checklist is purposefully more generalized than the one from the Sankaran et al^33^ publication as it is meant to be specific enough to serve as a practical roadmap yet generic enough to be applicable to varying organizational cultures and project scenarios. Application of each strategy in our case is specified in Table 4.^10^^,^^11^^,^^33^, ^34^, ^35^, ^36^

**Table 3:** Health Care Novel Technology Change Management (CM) Strategies Checklist

**Strategy 1: Assemble project team with necessary expertise**	☐ Set up a core project team. ☐ Incorporate additional team members with appropriate expertise on the basis of project phase-specific needs.
**Strategy 2: Anticipate potential institutional cultural and regulatory hurdles**	☐ Align team’s understanding of institutional culture and regulations regarding new technology CM. ☐ Devote effort at the beginning of project to predict possible hurdles and discuss potential preventive strategies and solutions. ☐ Procure leadership support and devise appropriate resources when navigating hurdles.
**Strategy 3: Add agility to project planning and execution**	☐ Summarize and review lessons learned from relevant past projects within and/or outside the institution to establish a starting point. ☐ Coordinate an agile mindset across the project team at the beginning and throughout the CM for planned and unplanned circumstances. ☐ Allocate ample time for execution.
**Strategy 4: Accommodate institutional culture and regulations**	☐ Orient CM efforts toward adding value to institution-specific missions. ☐ Assess level of trust toward new technology-related changes in your institution on the basis of recent past experiences. ☐ Consider creative approaches to address native CM needs.
**Strategy 5. Early clinical partner buy-in and stakeholder engagement**	☐ Initiate and maintain in-depth conversations with clinical leaders regarding the technology and the change underway. ☐ Persuade a few clinical leaders to be advocates of the change. ☐ Partner closely with clinical partners throughout the CM.
**Strategy 6: Consistent Communication**	☐ Consider tiered communication regarding the change underway: starting from among project team, to clinical leaders, and uniformly from clinical leaders to all clinical partners. ☐ Consider creating a project-specific frequently asked questions that is accessible to staff and that is updated as questions arise during the CM process. ☐ Empower new technology CM internally and externally by sharing your experience via creditable platforms (eg, professional conferences).

**Table 4:** How Our Case Had Applied the Strategies

**Strategy 1: Assemble project team with necessary expertise**	The project recruited team members who were knowledgeable experts, self-driven and proactive in defining and executing tasks. Experts were brought in to assist certain aspects of the project when needed. The expertise applied included but was not limited to health care systems engineering research, Institutional Review Board (IRB) approval process, information technology (IT), legal, contracts and data sharing, patient electronic health record and database management, specialized project management in health care technology management, third-party risk management, surgical systems operations, and supply chain management. The project enlisted a project manager (PM) with inter-institutional data sharing expertise ensured the task completion timeliness and reported project statuses periodically to governing bodies. Relevant contracts were created and updated with intention for re-use in future contract negotiations or expansion to more operating rooms (OR). Another PM with successful prior experience with health care technology implementation led the CM.^34^ The PM accomplished many formal, informal and behind-the-scenes effective communications with the OR staff, unions, and managers of nursing and anesthesia to ensure that they were informed of and were engaged in the project progress to create advocates for the project. This PM and the researchers also actively engaged the institution’s legal department, IRB, and media relations in preparation for the event if the project was publicized.
**Strategy 2: Anticipate potential institutional cultural and regulatory hurdles**	The project team anticipated and addressed various privacy and data integrity concerns from the approval committees by collaborating with in-house risk management; IT and infrastructure proponents to create data safety protocols to satisfy international agreements; and legal, employer, staff, and patient confidentiality requirements. A surgeon core team proponent delivered numerous presentations to committees clarifying the capability and safeguards of the system. The team’s use of briefing documents and preliminary discussions with committees facilitated the familiarization of the transformational and innovative nature of the technology. These efforts allowed the project team to address the questions that the committees posed and to minimize misperceptions and misinterpretations. The team minimized potential OR staff resistance by working with surgical leadership to reinforce the consistent message that data collected from this system would not be used for individual performance assessment, adherence, or disciplinary purposes, but rather for OR safety, quality, efficiency, and teamwork enhancement. They also verified that under state law the videos were not discoverable for legal purposes.^35^
**Strategy 3: Add agility to project planning and execution**	The project team deliberated a strategy and executed a CM plan that were both predictive and agile. The plan’s predictability took inspiration from existing CM models (Table 1) with added experience from a recent successful internal CM project.^34^ The team maintained an agile mindset to accommodate for an unconventional hybrid (research and practice) approval process. To satisfy committee inquires of data ownership and governance, the team allowed ample time to work closely with approval committees, legal, contracting, and the internal IT teams to establish that the surgeon whose case was recorded as well as the institution would own the data.
**Strategy 4: Accommodate institutional culture and regulations**	A vital component of this project’s strategy was customizing to the culture and complexity of our institution. For example, the project team aligned the hybrid project approval pathway with greater affinity toward research to leverage existing data security approval pathways that existed within the research enterprise. It was also important that the team allocated appropriate time, resources and expertise to follow the appropriate committee approval sequence without “shortcuts,” which largely facilitated the timeliness of project delivery. These efforts were convincing to leadership that this project was well aligned with the institution’s primary value “the needs of the patients come first,” which facilitated the project’s navigation through the institution’s intricate and unique committee approval process. Different institutions may experience different levels of hesitation and trust toward new technology and toward change, depending on its prior new technology implementation and CM experiences. Lessons learned from recent internal CM projects involving updated technology albeit of different scale and nature of the technology^34^^,^^36^ contributed tremendously to this implementation, especially the CM process. It is worth noting that the salary structure and level of employee unionization involved in the CM may matter in implementation. All physicians in our institution are salaried as are many of the allied health groups. However, select OR staff groups are unionized, which prompted the project team’s early engagement and communication with unions for their buy-in.
**Strategy 5. Early clinical partner buy-in and stakeholder engagement**	It was decided at the beginning of the project that an important aspect to facilitate CM was early stakeholder engagement and buy-in.^34^ Effective leadership was recognized as a central element and necessary resource during change particularly to address employee resistance. Thus, the team included committed leaders from surgical leadership, in which the change was to take place, to actively support the project activities. These leaders provided generous and sustained support within the scope of their normal day-to-day leadership roles and in project-required activities (eg, committee approval process, CM, and system installation). The senior leaders’ involvement, visibility, and commitment from the project’s beginning sent a strong message across the enterprise that they would actively support this change with employees. Besides surgical leadership support and endorsement, 2 surgeons became core project team members and advocated for the implementation of the project among their colleagues and institutional leadership. This physician–research–administrator partnership led the core team and lent a respected and credible voice to CM activities among project stakeholders. Additionally, to de-freeze the existing OR culture,^10^^,^^11^ the project team invited key stakeholders (ie, nursing leadership) to attend the annual Surgical Safety Network Conference, 2021. Gaining deeper understanding of the OR Blackbox technology, hearing experiences shared by experienced institutions, and networking with institutions with similar interests via the conference were essential in boosting stakeholder buy-in and engagement. The conference addressed potentially negative preconceptions of the technology; and the multi-institutional collaborations developed among surgeons, anesthetists, and OR nurses. Shortly after the conference, these key stakeholders identified “influencers” and “resisters” in their department and the team worked closely to create advocates for the project and to minimize resistance.
**Strategy 6: Consistent Communication**	Communication to approval committees and OR staff regarding the project implementation was a concerted and controlled effort throughout the project. This information was only circulated among project team members during Phase 1 (Project team assembly), and among approval committees during Phase 2 (Committee approvals) before finally being communicated as uniformly and consistently as possible to OR staff members and other employees of the institution in Phase 3 (CM). Surgeon team members conducted presentations to and led discussions among approval committees regarding the project goals and plans. The nonpunitive nature of the technology and data security and patient privacy assurance were presented by a surgeon working in one of the 3 ORs in which the system was to be installed. The ease of peer-to-peer conversation among clinicians facilitated the approval process. OR staff were encouraged, but not forced, to learn about or to adopt the AI technology. Two important documents were created to socialize the project before it went live: the frequently asked questions (FAQ), and the “key message.” Staff were provided with resources to the technology and this project from, eg, the key message and frequently asked questions (FAQ). The FAQ contained 53 questions that were categorized to “why,” “what,” “who,” “where,” “when,” and “how” groups. It was published on the institution’s intranet and served as a resource for all employees who had questions about the project. It was inspired by FAQs published by other institutions using the system and was based on a successful internal example.^33^ It incorporated numerous rounds of feedback from the project team, surgery, nursing, and anesthesia leadership. It remains an active document with ongoing edits based on new information, comments, and concerns that arise. By creating and sharing this repertoire of resources to OR and all other hospital staff, the investigators hoped to encourage organization-wide awareness, as well as open and continuous conversations regarding AI technologies and their application. A quick response (QR) code is installed on the doors of the ORs where the system was installed that links to the FAQ. The key message is a one-page “elevator speech” for the project, sent by managers to relevant OR staff. It was intended to be the one and only message to be disseminated to all relevant OR staff at the same time regarding the launch of the project. The message was intended to be concise but contain the core information of the purpose and goals of the project, and the resources to obtain more information (eg, web link to the FAQ). Besides the FAQ and the Key Message, a patient-oriented standard script was created. It was intended to be used by OR staff to provide patients with adequate information and consistent answers if they noticed door signage about the presence of the system required by the IRB when being wheeled into the OR. To date, this patient script has not been needed. Additionally, a dedicated email address to which staff could send concerns was created. A shared document repository site was set up early in the process to facilitate communication within the project team. Additionally, the core team discussed project progress weekly at hour-long meetings; leads of expert teams hosted regular summaries meetings per their particular goals with their stakeholders. Weekly email updates were sent by the PM to core team and stakeholders highlighting key items: high-level milestones, contracts, committees, facilities, infrastructure and hardware, operational activities, and CM. The authors intended to empower broader artificial intelligence (AI) application in the organization by setting a positive and leading example of AI technology implementation in the highly confidential and private OR. The positive outcomes of the implementation in enhancing surgical practice, efficiency, and teamwork, enabling interdisciplinary research, and strengthening intraoperative and postoperative learning contributed to sustaining the acceleration of AI technology implementation within the organization. In addition, core project team members have shared our implementation and CM experiences, success stories, and lessons learned internally and externally via educational seminars to inspire relevant education and collaborative research. The team has also kept a continuous conversation with the AI technology company regarding questions that emerged from system usage and suggestions for system and technology improvement.

**Strategy 1: Assemble project team with necessary expertise**

• Set up a core project team.
• Incorporate additional team members with appropriate expertise on the basis of project phase-specific needs.

**Strategy 2: Anticipate potential institutional cultural and regulatory hurdles**

• Align team’s understanding of institutional culture and regulations regarding new technology CM.
• Devote effort at the beginning of the project to predict possible hurdles and discuss potential preventive strategies and solutions.
• Procure leadership support and devise appropriate resources when navigating hurdles.

**Strategy 3: Add agility to project planning and execution**

• Summarize and review lessons learned from relevant past projects within and/or outside the institution to establish a starting point.
• Coordinate an agile mindset across the project team at the beginning and throughout the CM for planned and unplanned circumstances.
• Allocate ample time for execution.

**Strategy 4: Accommodate institutional culture and regulations**

• Orient CM efforts toward adding value to institution-specific missions.
• Assess level of trust toward new technology-related changes in your institution based on recent past experiences.
• Consider creative approaches to address native CM needs.

**Strategy 5. Early clinical partner buy-in and stakeholder engagement**

• Initiate and maintain in-depth conversations with clinical leaders regarding the technology and the change underway.
• Persuade a few clinical leaders to be advocates of the change.
• Partner closely with clinical partners throughout the CM period.

**Strategy 6: Consistent communication**

• Consider tiered communication regarding the change underway: starting from among project team, to clinical leaders, and uniformly from clinical leaders to all clinical partners.
• Consider creating a project-specific frequently asked questions (FAQ) that is accessible to staff and that is updated as questions arise during the CM process.
• Empower new technology CM internally and externally by sharing your experience via creditable platforms (eg, professional conferences).

## Results

### Case Study Outcomes

The project stayed on budget and on time despite the ongoing coronavirus disease 2019 pandemic. The CM effort was deemed successful by project team members, stakeholders, and OR staff: the team received mostly positive user feedback; only 3 out of hundreds of OR staff who work in the ORs where the system is installed opted out. There has not been any report of staff dissatisfaction through the feedback mechanism after go-live. By the time that article was written, which was approximately 7 months after the implementation, 2 of the 3 staff who opted out had opted back in. For staff who joined after the system implementation, a preceptor dedicates time to review the information regarding the system with new staff.

This transformational CM created evidential rippling effect from the 3 ORs that implemented the system to the other parts of the organization. For example, other surgical specialties such as the trauma and critical care/emergency department have initiated requests for the system to be fitted in their ORs. The system has also facilitated unexpected research initiative; for example, a patient position project in the nursing department was inspired and made possible by this system. The usability and effectiveness (eg, staff feedback) of this technology and its effect on practice improvement are being studied. Initially designed to support the department of surgery, other departments such as anesthesiology and nursing have expressed interest in using the system to conduct research studies.

### CM Model for Novel Technology Implementation in the OR

During the project initiation phase, we conducted literature review to gain an understanding of and to draw inspiration from existing CM models. As we predicted the challenges of our CM, we realized that no existing model addressed our project’s transformational CM needs adequately as they are often applied as guiding, supportive, and/or complimentary frameworks for changes within the complex and adaptive health care system.^37^ Additionally, our CM experienced unexpected challenges besides expected ones. For example, stakeholders who were not members of the approval committee inserted themselves into the committee approval process. We saw and used it as an opportunity to share knowledge regarding AI technology application, accelerate institutional AI technology adoption, and initiate interdepartmental relationships.

We summarized experiences and findings from this CM process and created a customized model: the Healthcare Novel Technology (HNT) CM model (Figure), and a checklist^33^ (Table 3) to guide future novel technology implementation in the OR. With the HNT CM model, we hoped to add value to the existing health care CM literature by creating a transformational CM journey map that provides steps to follow and room to customize for health care organizations to leverage. We intended to present our model in a practical and relatively simple manner so that it would be easy to understand and to apply by future users, although inspired by navigating the complex novel technology CM in the OR.

**Figure:**
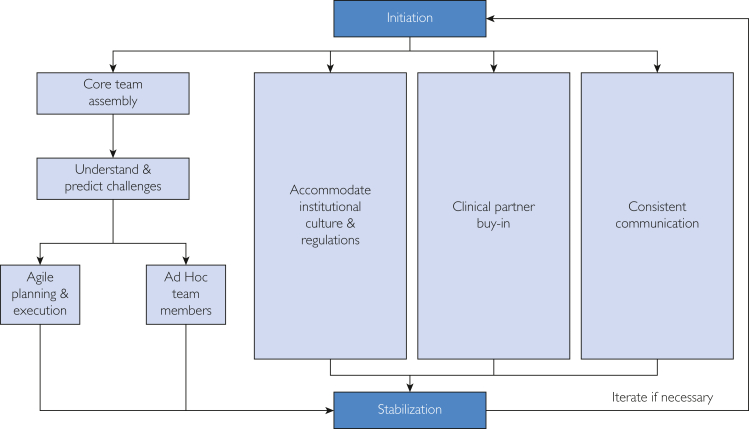
The Healthcare Novel Technology Change Management (HNT CM) model.

## Discussion

Before initiations of this AI–enabled system implementation, de-freezing^10^^,^^11^ of the existing culture started taking place when the authors initiated in-depth conversations with surgical leaders to gain understanding of the current surgical practice, familiarizing them with the AI technology and its reception in hospitals in the United States and internationally. Recognizing that nursing and anesthesia staff collaborate with the surgeons, the authors included the nursing and anesthesia leaders in the conversation by inviting them to attend the Surgical Safety Network conference, in which the technology inventors and hospitals that have implemented the system spoke extensively about the technology, implementation experiences and outcomes. As the leaders established greater understanding and trust of the technology, they became advocates who willingly shared their knowledge and enthusiasm with colleagues later when the project progressed to the stage of strategized communication to further de-freeze the existing culture.

OR staff were encouraged but not forced to learn about or to adopt the AI technology. Staff were provided with resources to the technology and this project from, for example, the key message and FAQ. They were provided the option to opt out of working in an OR in which the system was implemented by contacting their supervisor to initiate a room reassignment or to email the opt-out request to an email box that was set up for this project and managed by the project team. This email box was dedicated to answering questions regarding the system to process opt-out requests and case review requests. Difficulties with staff in older generations before the AI implementation were not detected. The 3 staff who opted out were all junior technicians.

The authors compiled questions raised during these periodic and ad hoc meetings among the project team members and the surgical, anesthesia, and nursing leaders and from emails received by the project email box into an FAQ. The FAQ incorporated questions and answers regarding the AI technology of the system as well as the purposes and expected outcomes of this project. It was then published on the organization’s intranet and updated periodically as new questions emerged. By creating and sharing this repertoire of resources to ORs and all other hospital staff, the authors hoped to encourage organization-wide awareness as well as open and continuous conversations regarding AI technologies and their application.

The investigators intended to empower broader AI application in the organization by setting a positive and leading example of AI technology implementation in the highly confidential and private OR. The positive outcomes of the implementation in enhancing surgical practice, efficiency, and teamwork; enabling interdisciplinary research; and strengthening intraoperative and postoperative learning contributed to sustaining the acceleration of AI technology implementation within the organization.

In addition, core project team members have shared our implementation and CM experiences, success stories, and lessons learned internally and externally via educational seminars to inspire relevant education and collaborative research. The team has also maintained continuous conversation with the AI technology company regarding questions that emerged from system usage and suggestions for system and technology improvement.

The IRB approval obtained by this project was for the AI–enabled audiovisual recording technology use in the OR and covers all phases of this project. In this IRB protocol that all key stakeholders contributed to create, staff were not to be consented due to the spontaneity and the number of OR entry by staff that are affiliated with but do not work in the OR, for example, pathology specialists. Staff can voluntarily opt out by contacting their supervisor or by emailing their request to the project email box. Those who chose to opt out were not required to provide a specific reason as specified in our IRB application. Other than opting out of an OR entirely, staff may request to opt out of a case on a specific date during a specific time range. We elected not to inquire opt-out reasons and had set up the email box to process the opt-out requests due to considerations of staff privacy.

Although conducting confidential surveys regarding staff satisfaction could contribute to quantitative measurement of CM outcomes, we did not do this because of an institutional survey moratorium at that time to address staff survey fatigue. It was also difficult to query OR staff because of their low email usage. As specified in our IRB application, our cross-functional team consensually selected the staff opt-out count as a quantitative measure of staff satisfaction. The low opt-out rate at the time of system implementation indicated staff acceptance of the system; the count at the “Stabilization” phase of the project (Figure) indicated high staff satisfaction. Two staff opting back in implied that system acceptance and satisfaction might have improved over time.

Requirement of resources, especially human resources, may be a limitation of the approach of our case. Specifically, a large cross-functional team of various expertise were a part of our CM project team at varied time ranges. It is important to note that the prioritization of this CM as well as much time and effort dedicated by clinical partners and leaders were toward voluntary engagement beyond their clinical hours as they saw the value of AI technology application in health care.

## Conclusion

We presented a successful CM case at our institution in which novel AI technology was implemented in the ORs for the purpose of advancing surgical care via enhanced and objective analysis of intraoperative performance. The implementation and CM aimed to create transformative innovation and practice impact for both patients and surgical staff. Inspired by this experience and previously established CM models, we created a new CM model, the “HNT CM” model, aiming to address the unique needs and characteristics of OR novel technology implementation. The HNT CM model may be adopted as a journey map to guide future similar processes; it may be customized and trialed to address individual institution and project’s needs and characteristics.

## Potential Competing Interests

The Mayo Clinic authors (Smith, Norasi, Herbst, Kendrick, Curry, Hallbeck, Cleary) certify that they have no affiliations with or involvement in any organization or entity with any financial or personal interest in the subject matter or materials discussed in this manuscript. T.P.G. is the founder and Director of Surgical Safety Technologies and equity holder of Surgical Safety Technologies, Inc. V.N.P. is Director of Analytics and equity holder at Surgical Safety Technologies, Inc. This article has not been published previously or has not been concurrently submitted for publication elsewhere.
